# *Zhezhoulinyphia* gen. nov. (Araneae, Linyphiidae) from Yunnan, China

**DOI:** 10.3897/zookeys.862.31406

**Published:** 2019-07-09

**Authors:** Muhammad Irfan, Gu-chun Zhou, Xian-jin Peng

**Affiliations:** 1 College of Life Sciences, Hunan Normal University, Changsha, Hunan 410081, China Hunan Normal University Changsha China

**Keywords:** Gaoligong Mountain, morphology, Linyphiinae, sheet web spiders, taxonomy

## Abstract

A new Linyphiinae genus *Zhezhoulinyphia***gen. nov.** from Yunnan Province is described together with its two new species: *Z.caperata***sp. nov.** (♂) and *Z.denticulata***sp. nov.** (♂, ♀). *Centromerusyadongensis* Hu & Li, 1987 is transferred to *Zhezhoulinyphia***gen. nov.** and a new combination, *Z.yadongensis* (Hu & Li, 1987), **comb. nov.** is proposed.

## Introduction

Linyphiidae Blackwall, 1859, the second largest spider family, contains 4591 species in 611 genera, which accounts for approximately 10% of the total number of spiders worldwide (WSC 2019). Li and Lin (2016) listed 371 Chinese linyphiid species in 154 genera, including 69 species in 16 genera in the subfamily Linyphiinae. Yunnan is a biodiversity-rich spot in south China and about 9 genera and 39 species of linyphiid spiders have been described from there (Wunderlich and Song 1994; Xia et al. 2001; Liu and Chen 2010; Zhao and Li 2014; Zhao and Li 2017; Irfan and Peng 2018).

While examining the specimens collected from Gaoligong Mountain, *Zhezhoulinyphia* gen. nov. with two new species were identified and are described here.

## Material and methods

Specimens were collected by hand picking and beating shrubs and were kept in 75% ethanol. The type specimens are deposited at the College of Life Sciences, Hunan Normal University, Changsha, China. After dissection, epigyna were cleared in trypsin enzyme solution before examination and photography. The left male palp was used for description and illustration. Specimens were examined, measured with a Leica M205C stereomicroscope. Photos were taken with a digital camera Canon PowerShot G12 mounted on an Olympus BX53 and Leica MC170 HD mounted on a Leica M205C. Stacked focus images were generated using Helicon Focus software (3.10, free). A map was created using ArcMap 10.2, and then modified using Adobe Photoshop CS2 Extended. Leg measurements are given in the following order: total length (femur, patella + tibia, metatarsus, tarsus). All measurements are given in millimeters (mm).

Abbreviations used in the text and figures are as follows:

**AE** anterior wall of epigyne;

**AER** anterior eyes row;

**ALE** anterior lateral eyes;

**AME** anterior median eyes;

**AME–AME** distance between AME;

**AME–ALE** distance between AME and ALE;

**CO** copulatory opening;

**CRL** cymbial retrolateral lobe;

**DPE** dorsal lobe of embolic plate;

**DSA** distal suprategular apophysis;

**E** embolus;

**EM** embolic membrane;

**FD** fertilization ducts;

**P** parmula;

**PC** paracymbuim;

**PER** posterior eyes row;

**PLE** posterior lateral eyes;

**PME** posterior median eyes;

**PME–PME** distance between PME;

**PME–PLE** distance between PME and PLE;

**PMP** posterior median plate;

**R** radix;

**RA** radical apophysis;

**S** spermathecae;

**ST** suprategulum;

**T** tegulum;

**W** wrinkle.

## Taxonomy

### Linyphiidae Blackwall, 1859

#### Zhezhoulinyphia gen. nov.

Taxon classificationAnimaliaAraneaeLinyphiidae

http://zoobank.org/1250FBD2-CA7E-4C9A-93C3-39C8A27A8F0E

##### Type species.

*Zhezhoulinyphiadenticulata* sp. nov.

##### Etymology.

The species name comes from the Chinese word “褶皱(Zhezhou)” meaning “wrinkle” and referring to parmula with wrinkles in epigyne. Gender feminine.

##### Diagnosis.

*Zhezhoulinyphia* gen. nov. resembles *Diplostyla* Emerton, 1882, *Kaestneria* Wiehle, 1956, *Laetesia* Simon, 1908 and *Laperousea* Dalmas, 1917 in: Epigyne with parmula originating from posterior margin of posterior median plate with a socket ventrally (Fig. 7A–D; Ivie 1969, figs 105–108; van Helsdingen 1972, fig. 8; Irfan and Peng 2018, figs 5C, D, 6C, D;). Distal margin of radix semicircular with teeth as in *Laetesia* and *Laperousea* (Figs 4A, B, 5C–E, 6A, B; Millidge 1988, figs 145–146). It can be distinguished by the following characters: the embolus and embolic membrane arise from the dorsal side of the distal margin of radix (Figs 4A, B, D, 5C–E, 6A, B), whereas this arises from the lateral (inner) side of the embolic plate in *Kaestneria* and *Laetesia* (Millidge 1988, figs 145, 149; Zhao and Li 2014, figs 47B, 49B) and in *Diplostylaconcolor* embolus arises near base of cymbium and extends parallel along with full length of cymbium (Ivie 1969, figs 107, 108); distal suprategular apophysis proximally broad with teeth, distal part strongly curved into inverse U-shaped, and almost touching distal margin of paracymbium (Figs 4A, B, D, 5G, 6A, B), whereas it is protruding upward with a notch in *Laetesia* (Millidge 1988, fig. 146); without a notch in *Kaestneria* (Tao et al. 1995, figs 77–78) and *Diplostylaconcolor* (Ivie, 1969, fig. 108); parmula extending towards anterior margin first then folding backward, distal part with transverse wrinkles (Figs 7A–D, 9A–D, 10A, B), whereas parmula not folded and without transverse wrinkles both in *Kaestneria* and *Laetesia* (Millidge 1988, figs 145, 149; Zhao and Li 2014, figs 47B, 49B) and in *Diplostylaconcolor*, epigyne parmula reduced, invisible in ventral view, ventral plate with very long and slender scape (Ivie, 1969, figs 105, 106).

##### Description.

Large sized, 4.0–5.5. Male cephalic region strongly elevated, ocular area with spines, extending forward. AER procurved, PER slightly recurved. Chaetotaxy: 2–2–2–2. TmI 0.9–1.3, TmIV 0.5–0.75. Leg formula I–II–IV–III. Legs yellow without obvious patterns.

Male Palp: Femur almost equal to the collective length of patella and cymbium (Fig. 5A, B). Patella shorter than tibia, dorsally with a long spine (Figs 5A, B, 6A, B). Tibia with three retrolateral and a dorsal trichobothria (Figs 4A, B, 5G, 6A, B). Cymbium conical flask-shaped, with a cymbial retrolateral lobe (Figs 4B, 5B, G, 6B). Paracymbium large, distal arm broad with round tip (Figs 4B, 5F, 6B). Distal suprategular apophysis sclerotized, proximal end broad with teeth, strongly curved distally (Figs 4A–C, 5G, 6A, B). Radix long, proximal end with finger-shaped dorsal lobe of embolic plate; distal margin semicircular with teeth and a radical apophysis. Embolus and embolic membrane arise from dorsal side of distal semicircular serrated margin of radix (Figs 4A–C, 5C–E, 6A).

**Figure 1.**
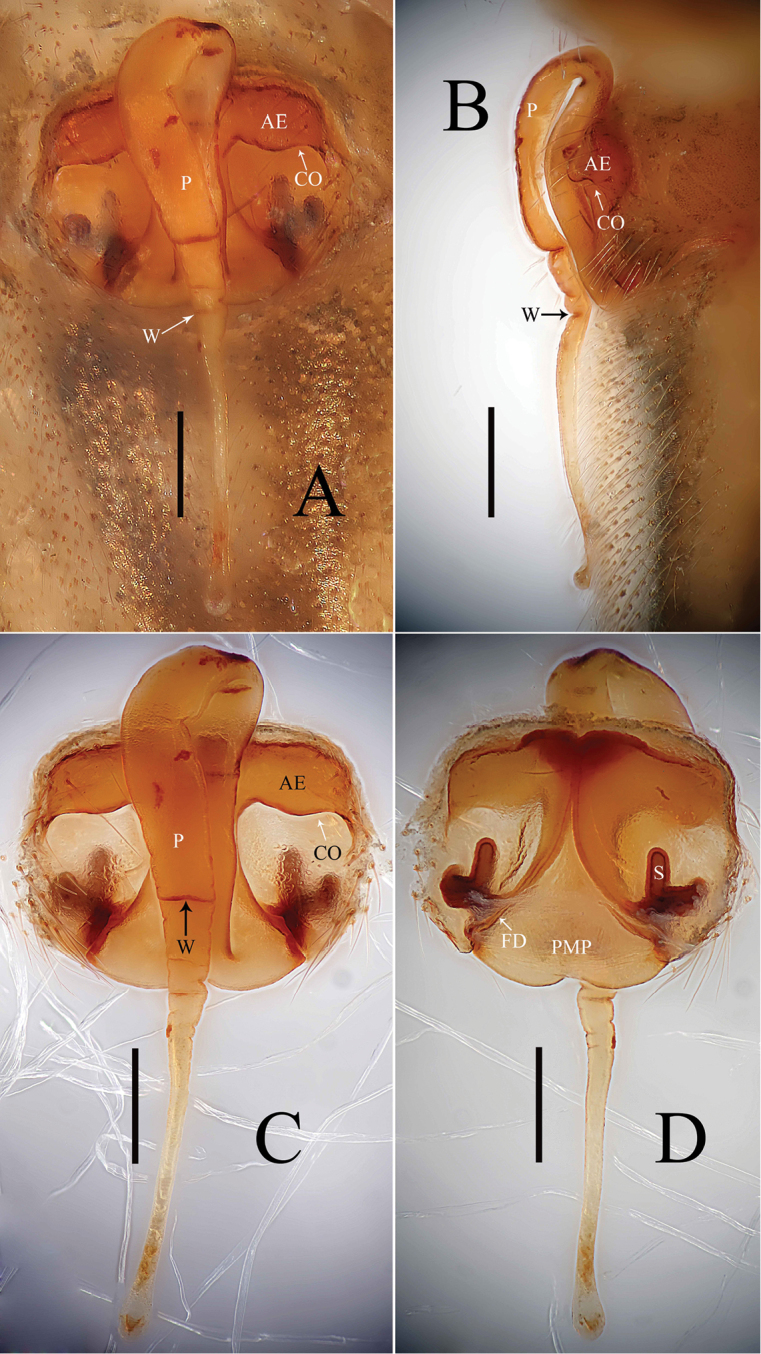
*Zhezhoulinyphiacaperata* sp. nov., female holotype **A** epigyne, ventral view (before dissected from the body) **B** ditto, lateral view **C** ditto, ventral view (after dissected from the body) **D** vulva, dorsal view. Scale bars: 0.2 mm (**A–D**).

**Figure 2.**
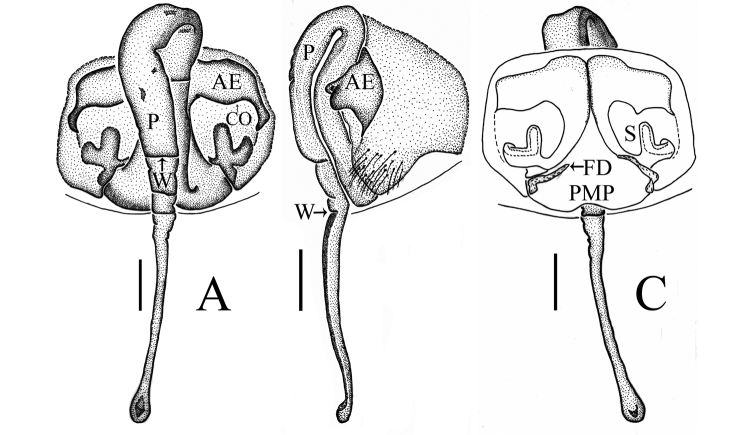
*Zhezhoulinyphiacaperata* sp. nov., female holotype **A** epigyne, ventral view **B** ditto, lateral view **C** vulva, dorsal view. Scale bars: 0.2 mm (**A–C**).

Epigyne (Figs 1A–D, 2A–C, 7A–D, 9A–D, 10A, B): Anterior wall of epigyne (AE) longer than wide, posterior margin with a big outgrowth; copulatory openings present inside atrium; parmula long, extending towards anterior margin first then folding backward, distal part with transverse wrinkles (number of wrinkles varies among different species), distal tip with a socket posteriorly. Vulva: posterior median plate cordiform; copulatory ducts long, arch-shaped; spermathecae U- or L-shaped, present mesally on posterior median plate; fertilization ducts long, extending mesally.

##### Distribution.

Yunnan, Tibet (China) (Fig. 12).

#### Zhezhoulinyphia caperata sp. nov.

Taxon classificationAnimaliaAraneaeLinyphiidae

http://zoobank.org/4C3946E0-30E2-48F4-B560-0B7BCF6D7704

Figs 1 , 2 , 3 , 12

##### Types.

Holotype female, **China, Yunnan Province**: Gongshan County, Bingzhongluo Township, Chukuai，27.97928°N, 98.47389°E，alt. 3725 m, 19 August 2006, Peng Hu (Hu060819). Paratypes: 2 females, Gongshan County, Dulongjiang Township, Maku, 27.68611°N, 98.29660°E, alt. 2097 m, 2 September 2006, Peng Hu (Hu060902).

##### Etymology.

The species name comes from the Latin adjective “*caperatus*”, meaning “wrinkled” and referring to parmula with wrinkles in epigyne.

##### Diagnosis.

*Zhezhoulinyphiacaperata* sp. nov. can be distinguished from *Z.denticulata* sp. nov. by having the anterior wall of epigyne wider than long, posterior margin without distinct outgrowth (Figs 1A–C, 2A, B), whereas it is longer than wide, with a big outgrowth in *Z.denticulata* sp. nov. (Figs 7A–D, 9A–D, 10A–C). Parmula with three transverse wrinkles in new species (Figs 1A–C, 2A, B), whereas there are seven to twelve in *Z.denticulata* sp. nov. (Figs 7A–D, 9A–D, 10A–C).

##### Description.

**Figure 3.**
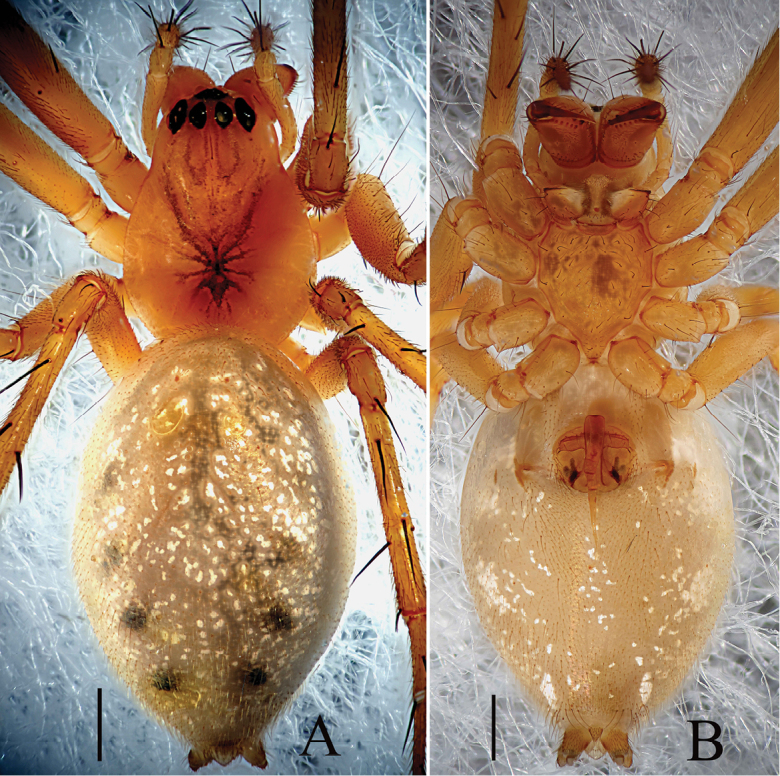
*Zhezhoulinyphiacaperata* sp. nov., habitus of female holotype **A** dorsal view **B** ventral view. Scale bars: 0.5 mm (**A–B**).

Female (holotype): Total length: 5.35. Carapace 1.97 long, 1.54 wide, yellow to reddish, cephalic region slightly elevated with spine like hairs (Fig. 3A, B), fovea, cervical and radial grooves distinct (Fig. 3A); Clypeus 0.48 high. Sternum longer than wide, light yellow, provided with spine-like hairs; labium wider than long; maxillae long, distal end broader, with hairs (Fig. 3B). Chelicerae with 6 promarginal and 3 retromarginal teeth. AER recurved, PER straight, slightly wider (Fig. 3A). Eye sizes and interdistances: AME, 0.11; ALE, 0.16; PME, 0.13; PLE, 0.14; AME-AME, 0.06; PME-PME, 0.10; AME-ALE, 0.09; PME-PLE, 0.13; AME-PME, 0.15; ALE-ALE, 0.66; PLE-PLE, 0.71; ALE-PLE contiguous. Length of legs: I, 9.55 (2.68, 2.97, 2.62, 1.28); II, 8.65 (2.49, 2.68, 2.33, 1.15); III, 6.03 (1.83, 1.81, 1.56, 0.83); IV, 7.40 (2.21, 2.27, 1.99, 0.93). Leg formula I–II–IV–III. TmI, 1.02 and TmIV, 0.68. Tibial dorsal spine formula: 2–2–2–2. Abdomen 3.38 long, 2.25 wide, oval, grey, mid-dorsally with a black pattern with irregular white patches extending laterally, ventral side pale with irregular white patches (Fig. 3A, B). Epigyne (Figs 1A–C, 2A, B): Anterior wall of epigyne (AE) wider than long, with wave-like margin posteriorly; copulatory openings present inside atrium; parmula long, extending towards anterior margin first then folding backward, distal part with 3 transverse wrinkles, distal tip with a socket posteriorly. Vulva: posterior median plate broad and cordiform. Copulatory ducts long, arch-shaped; spermathecae L-shaped, present mesally on posterior median plate; fertilization ducts long and extending mesally.

##### Male.

Unknown.

##### Distribution.

China (Yunnan) (Fig. 12).

#### Zhezhoulinyphia denticulata sp. nov.

Taxon classificationAnimaliaAraneaeLinyphiidae

http://zoobank.org/CC3698D0-721A-4DB3-88F8-7CCB156FA036

Figs 4 , 5 , 6 , 7 , 8 , 9 , 10 , 11 , 12

##### Types.

Holotype male, **China, Yunnan**: Fugong County, Lishadi Township, Shibali, 27.10520°N, 98.77980°E, alt. 2530 m, 10 August 2005, Guo Tang (Tang–05–02). Paratypes: 2 males 2 females, same data as holotype (Tang–05–02); 2 females, Fugong County, Pihe Township, Yueliangtian Village, 26.56784°N, 98.90884°E, alt. 1520 m, 24 August 2005, Guo Tang (Tang–05–08); 6 males 1 female, Baoshan City, Nankang Yakou (National 320 Road), 24.43717°N, 98.46054°E, alt. 2186 m, 30 October 2003, Guo Tang (Tang031030); 1 female, Gongshan County, Qiqi Dongshaofang, 27.69521°N, 98.48514°E, alt. 3208 m, 29 September 2007, Xian-jin Peng (20071001); 1 female, Gongshan County, Qiqi Dongshaofang, 27.69521°N, 98.48514°E, alt. 3208 m, 29 September 2007, Xian-jin Peng (20070929); 1 female, Gongshan County, Cikai Township, Dabadi troops place north bank of Pula river, 27.78333°N, 98.51667°E, alt. 3030 m, 28 September 2002, Heng-mei Yan (Yan020928); 6 males 1 female, Nujiang Prefecture, Nujiang State Nature Reserve, No.12 bridge Camp area, 16.3 air km W of Gongshan, 27.71503°N, 98.50244°E, alt. 2775 m, 15–19 July 2000, Heng-mei Yan, D. H. Kavanaugh, Charles Griswold, Hong-bin Liang, Darrell Ubick and Da-zhi Dong (00–QD).

##### Etymology.

The species name comes from the Latin adjective “*denticulus*”, meaning “teeth” and referring to the distal suprategular apophysis (DSA) with teeth in male palp.

##### Diagnosis.

*Zhezhoulinyphiadenticulata* sp. nov. can be distinguished from *Z.caperata* sp. nov. by having the anterior wall of epigyne longer than wide, with a big outgrowth (Figs 1A–C, 2A, B), whereas wider than long, posterior margin without distinct outgrowth in *Z.caperata* sp. nov. (Figs 7A–D, 9A–D, 10A–C). Parmula with seven to twelve transverse wrinkles in new species (Figs 1A–C, 2A, B), whereas there are three in *Z.caperata* sp. nov. (Figs 7A–D, 9A–D, 10A–C).

##### Description.

**Figure 4.**
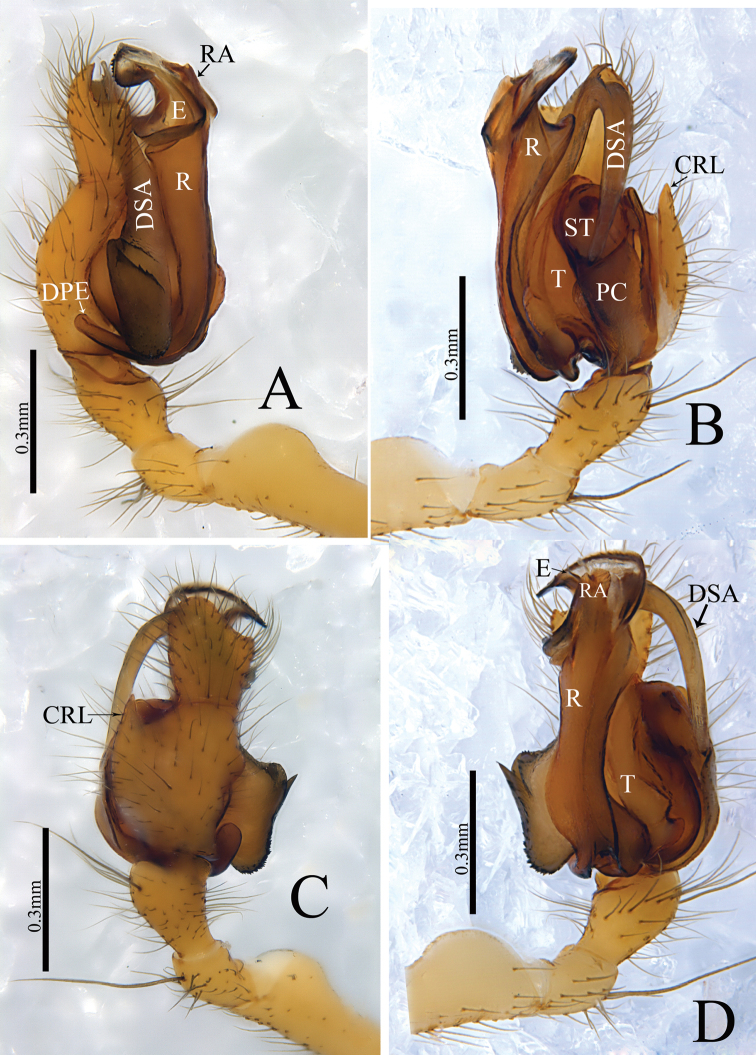
*Zhezhoulinyphiadenticulata* sp. nov., male holotype, left palp **A** prolateral view **B** retrolateral view **C** dorsal view **D** ventral view. Scale bars: 0.3 mm (**A–D**).

**Figure 5.**
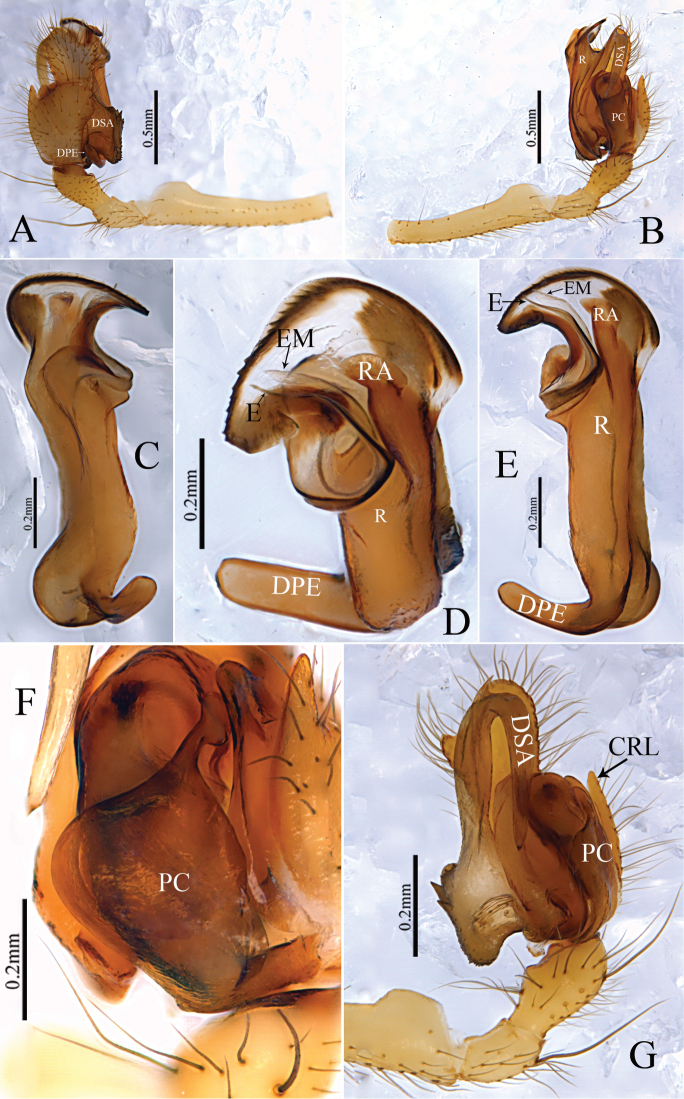
*Zhezhoulinyphiadenticulata* sp. nov. **A, B** male holotype, left palp (Tang-05-02) **C–G** one of paratype male, left palp (00-QD).

**Figure 6.**
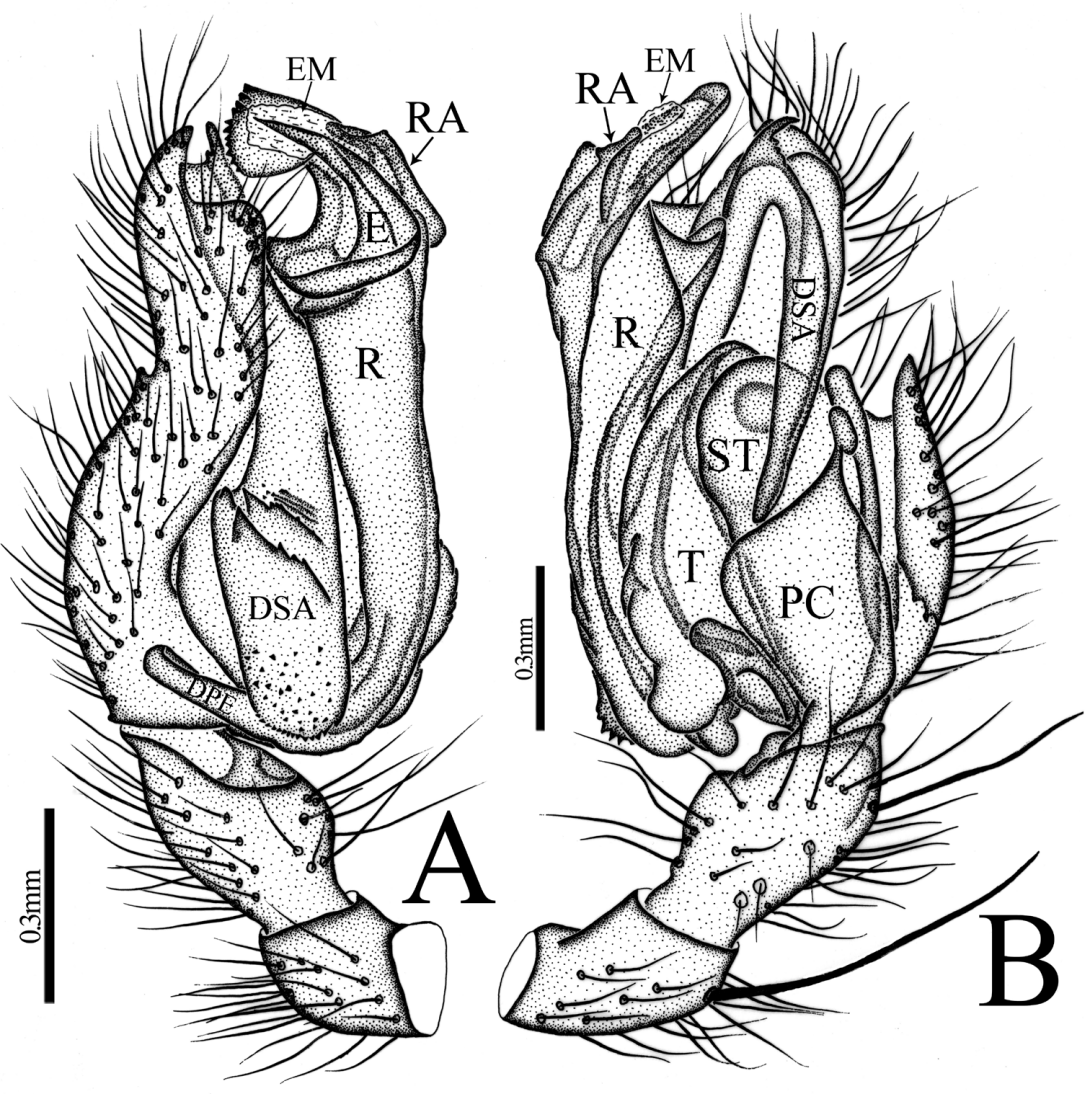
*Zhezhoulinyphiadenticulata* sp. nov., male holotype, left palp **A** prolateral view **B** retrolateral view. Scale bars: 0.3 mm (**A–B**).

Male (holotype): Total length: 4.24. Carapace 1.80 long, 1.43 wide, yellow, cephalic lobe 0.91 long, fovea, cervical and radial grooves distinct (Fig. 8A, B); Clypeus 0.59 high (Fig. 8B). Sternum longer than wide, brown, with spine-like hairs; labium wider than long; maxillae long, distal end broader with hairs (Fig. 8C). Chelicerae with three promarginal and three retromarginal teeth. AER recurved, PER straight, slightly wider. Eye sizes and interdistances: AME, 0.07; ALE, 0.10; PME, 0.11; PLE, 0.09; AME-AME, 0.07; PME-PME, 0.08; AME-ALE, 0.14; PME-PLE, 0.15; AME-PME, 0.16; ALE-ALE, 0.55; PLE-PLE, 0.59; ALE-PLE, 0.02. Length of legs: I, 12.63 (3.38, 3.88, 3.77, 1.60); II, 11.04 (3.16, 3.33, 3.23, 1.32); III, 7.21 (2.16, 2.16, 2.05, 0.84); IV, 8.93 (2.59, 2.63, 2.69, 1.02). Leg formula I–II–IV–III. TmI 1.25 and TmIV 0.74. Tibial dorsal spine formula: 2–2–2–2. Abdomen 2.44 long, 1.19 wide, cylindrical, grey, mid dorsally with a grey pattern and densely covered with white patches extending laterally, ventral side dark grey (Fig. 8A–C). Palp (Figs 4A–D, 5A–G, 6A, B); femur almost equal to collective length of patella and cymbium, distally expanded, dorsally with a row of fine spine like hairs (Fig. 5A, B); patella shorter than tibia, dorsally with a long spine (Fig. 5A, B, 6A, B); tibia conic, with three retrolateral and a dorsal trichobothria, dorsally with a long spine (Figs 4A, B, 5G, 6A, B); cymbium conical, flask shaped, with a cymbial retrolateral lobe protruding upward (Figs 4B, 5B, G, 6B); paracymbium sclerotized, simple, distal arm longer than wide, tip pendulum-shaped (Figs 4B, 5F); distal suprategular apophysis proximally broad with teeth, distal part strongly curved into inverse U-shaped, and almost touches distal margin of paracymbium. Embolic division: dorsal lobe of embolic plate long, sclerotized, overlapping cymbium (Figs 4A, 5C–E, 6A); radix long, distal margin semicircular with teeth (Figs 4A, B, 5C–E, 6A, B); radical apophysis sclerotized, with blunt end (Figs 4A, B, 5C–E, 6A, B); embolic membrane and embolus arise from the dorsal margin of semicircular part (Figs 4A, B, 5C–E, 6A, B); embolus sclerotized, long, curved and almost touches serrated margin of radix (Figs 4A, 5C–E, 6A).

**Figure 7.**
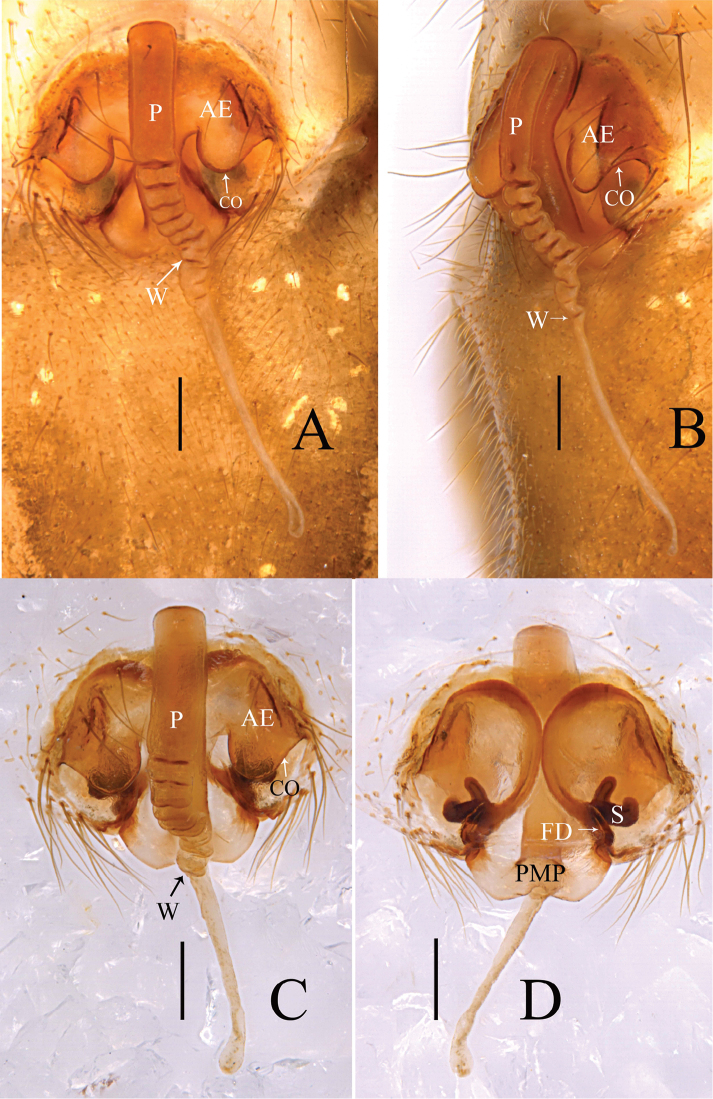
*Zhezhoulinyphiadenticulata* sp. nov., female (one of paratype, Tang–05–02) **A** epigyne, ventral view (before dissected from the body) **B** ditto, lateral view **C** ditto, ventral view (after dissected from the body) **D** Vulva, dorsal view. Scale bars: 0.2 mm (**A–D**).

**Figure 8.**
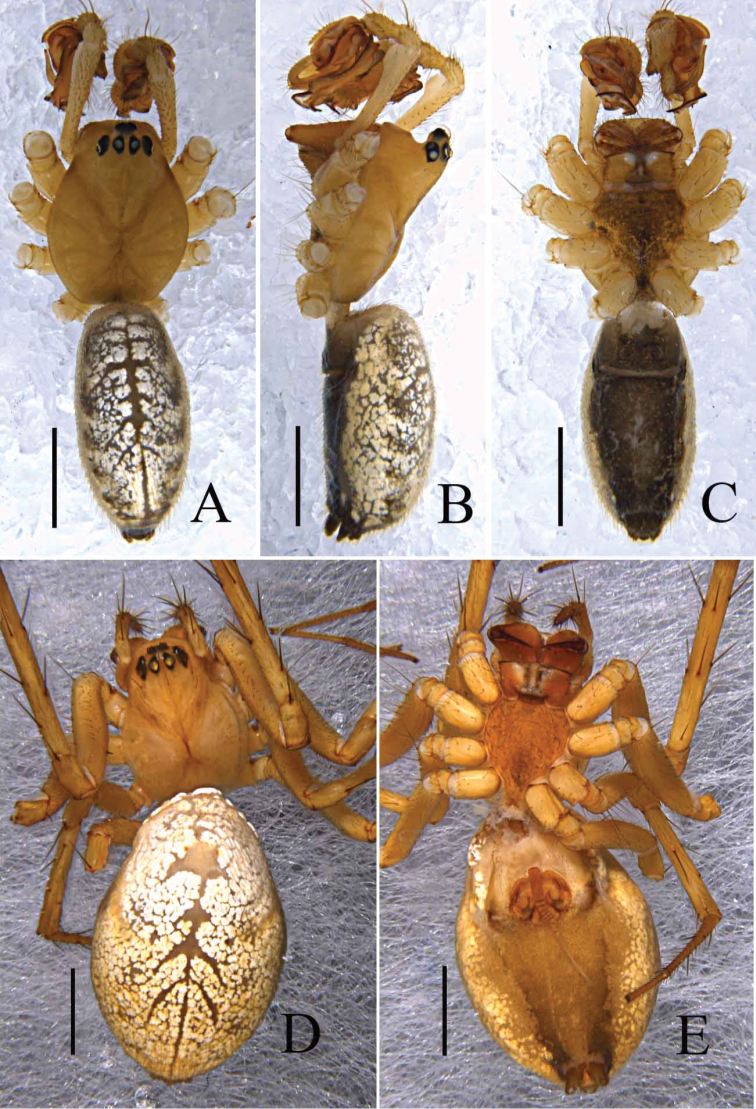
*Zhezhoulinyphiadenticulata* sp. nov., habitus of male holotype (**A–C**) and female, (one of paratypes, Tang–05–02; **D–E**) **A, D** Dorsal view **B** Lateral view **C, E** Ventral view. Scale bars: 1 mm (**A–D**).

**Figure 9.**
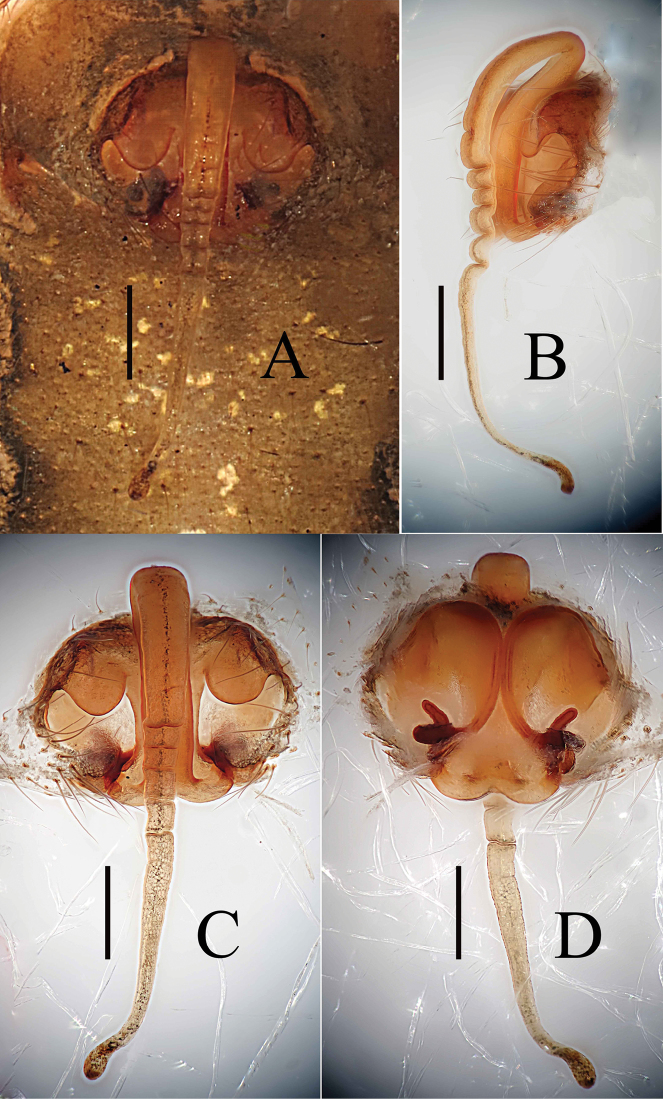
*Zhezhoulinyphiadenticulata* sp. nov., female (one of paratype, Tang–05–08) **A** epigyne, ventral view (before dissected from the body) **B** ditto, lateral view **C** ditto, ventral view (after dissected from the body) **D** vulva, dorsal view. Scale bars: 0.1 mm (**A–D**).

**Figure 10.**
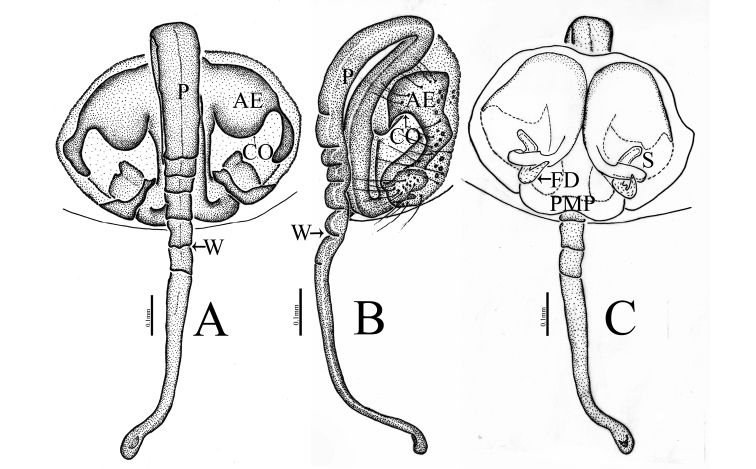
*Zhezhoulinyphiadenticulata* sp. nov., female (one of paratype, Tang–05–08) **A** epigyne, ventral view **B** ditto, lateral view **C** vulva, dorsal view. Scale bars: 0.1 mm (**A–D**).

**Figure 11.**
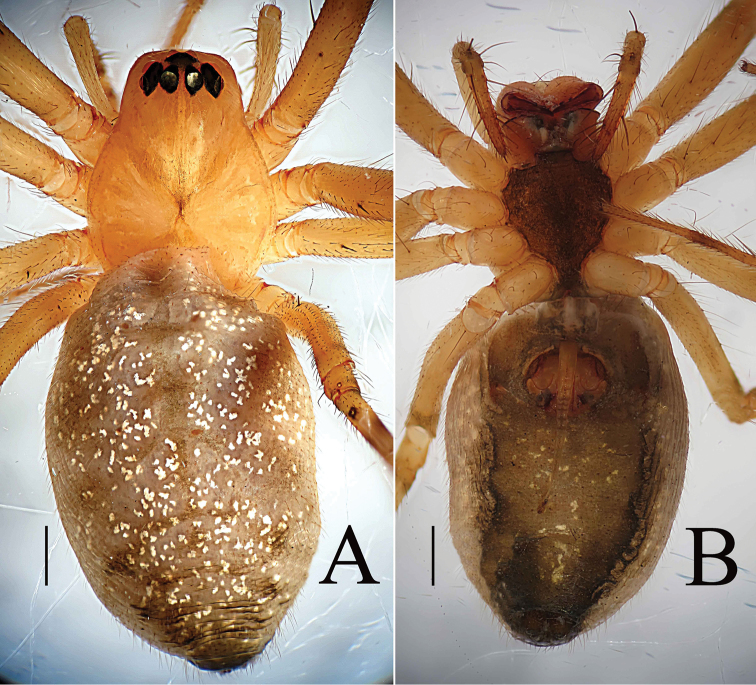
*Zhezhoulinyphiadenticulata* sp. nov., habitus of female (one of paratypes, Tang–05–08) **A** dorsal view **B** ventral view. Scale bars: 1 mm (**A–D**).

Female (one of paratype, Tang–05–02): Total length: 4.46. Carapace 1.67 long, 1.46 wide, yellow, cephalic region slightly elevated with spine like hairs, fovea, cervical and radial grooves distinct (Figs 8A, 11A). Clypeus 0.59 high. Sternum longer than wide, light orange, with spine-like hairs; labium wider than long; maxillae long, distal end broader with hairs (Figs 8B, 11B). Chelicerae with three promarginal and three retromarginal teeth. AER recurved, PER straight, slightly wider. Eye sizes and interdistances: AME, 0.09; ALE, 0.15; PME, 0.12; PLE, 0.12; AME-AME, 0.05; PME-PME, 0.08; AME-ALE, 0.07; PME-PLE, 0.12; AME-PME, 0.11; ALE-ALE, 0.59; PLE-PLE, 0.63; ALE-PLE contiguous. Length of legs: I, 9.67 (2.68, 3.09, 2.65, 1.25); II, 8.75 (2.59, 2.76, 2.35, 1.05); III, 6.31 (1.94, 2.01, 1.57, 0.79); IV, 7.37 (2.23, 2.24, 2.04, 0.86). Leg formula I–II–IV–III. Tm I, 0.92 and Tm IV, 0.53. Tibial dorsal spine formula: 2–2–2–2. Abdomen 2.79 long, 1.64 wide, oval, grey, mid dorsally with a grey pattern and densely covered with white patches extending laterally, ventral side brown (Figs 8A, B, 11A, B). Epigyne (Figs 7A–D, 9A–D, 10A, B): Anterior wall of epigyne (AE), longer than wide, posterior margin with a big outgrowth; copulatory openings present inside the atrium; parmula long, extending towards anterior margin first then folding backward, distal part with seven to twelve transverse wrinkles (Figs 7A–D, 9A–D, 10 A, B), distal tip with a socket posteriorly. Vulva: posterior median plate broad, cordiform; copulatory ducts long, arch-shaped; spermathecae U-shaped or L-shaped, present mesally on the posterior median plate; fertilization ducts long and extending mesally (Fig. 7D).

##### Distribution.

**Figure 12.**
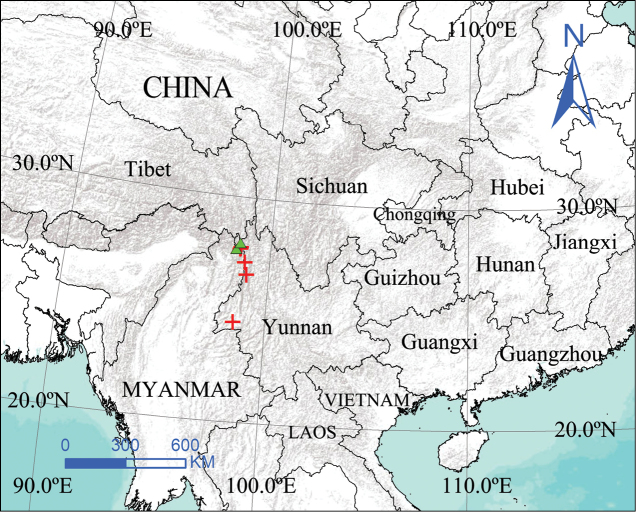
Distribution of *Zhezhoulinyphiacaperata* sp. nov. (triangle) *Zhezhoulinyphiadenticulata* sp. nov. (cross).

China (Yunnan) (Fig. 12).

#### Zhezhoulinyphia yadongensis

Taxon classificationAnimaliaAraneaeLinyphiidae

(Hu & Li, 1987) comb. nov.

Centromerus yadongensis Hu & Li, 1987: 343, fig, 21.1–4 (Df); Hu 2001: 494, fig. 328.1–3 (f).

##### Remarks.

The epigyne of *Centromerusyadongensis* Hu & Li, 1987 shares the similar characters to the genus *Zhezhoulinyphia* gen. nov. such as: parmula extending towards the anterior margin first then folding backward, the distal part with transverse wrinkles, whereas it can be distinguished from *Zhezhoulinyphiacaperata* sp. nov. and *Z.denticulata* sp. nov. by the number of the wrinkles in the parmula and the position of spermathecae (Figs 1, 2, 7, 9, 10; Hu 2001, figs 8–328). Based on the above-mentioned characters *Centromerusyadongensis* Hu & Li, 1987 is transferred to the genus *Zhezhoulinyphia* as *Z.yadongensis* com. nov. (Hu & Li, 1987).

##### Distribution.

China (Tibet) (WSC 2019).

## Discussion

*Zhezhoulinyphia* gen. nov. can be identified as the member of the subfamily Linyphiinae by the following morphological characters: female palp with claw; male palp without tibial apophysis; maxillae long and parallel; all the tibiae with two dorsal spines (Merrett 1963).

## Supplementary Material

XML Treatment for Zhezhoulinyphia

XML Treatment for Zhezhoulinyphia caperata

XML Treatment for Zhezhoulinyphia denticulata

XML Treatment for Zhezhoulinyphia yadongensis
